# Hypoxia‐induced endothelial dysfunction: Could targeting oxidative stress provide protection?

**DOI:** 10.1113/EP091276

**Published:** 2023-05-26

**Authors:** Gabriella M. K. Rossetti, Samuel J. Oliver, Aamer Sandoo, Jamie H. Macdonald

**Affiliations:** ^1^ Centre for Integrative Neuroscience and Neurodynamics (CINN) University of Reading Reading UK; ^2^ School of Human and Behavioural Sciences Bangor University Bangor UK

## 1 INTRODUCTION

Hypoxia can cause disturbances to vascular homeostasis and endothelial dysfunction and is believed to contribute to the pathophysiology of atherosclerosis. Over the past 10 years, a growing body of evidence implicates oxidative stress and diminished NO bioavailability as a key mechanistic pathway to explain the deleterious effects of acute and chronic hypoxia on endothelial function. In this connections article, we highlight three key papers that combine to characterize the effect of hypoxia on endothelial function, support the mechanistic role of oxidative stress and NO bioavailability, and set the scene for targeted interventions to prevent endothelial dysfunction in the presence of hypoxia.

The endothelium is a heterogeneous tissue for structure and function; therefore, a comprehensive assessment of endothelial function is crucial to understand the significance of hypoxia‐induced endothelial dysfunction. In the first (and, to date, only) article to examine the effect of hypoxia on microvascular and large‐vessel endothelial function in the same study, we observed a hypoxia‐induced reduction in endothelium‐dependent microvascular function (43% reduction in the perfusion response to acetylcholine) and endothelium‐dependent large vessel vasodilatation [18% reduction in flow‐mediated dilatation of the brachial artery (FMD)] (Jones et al., 2021). Notably, the extent of the decrease was approximately twofold higher in the microcirculation compared with the large vessels. This finding is interesting in the context of an oxidative stress model of hypoxia‐induced endothelial dysfunction, because the microvasculature is generally more sensitive to oxidative stress than large blood vessels. This is attributable to a greater surface area‐to‐volume ratio, making it more exposed to reactive oxygen species (ROS), which can cause oxidative stress. Furthermore, a greater prevalence of adhesion molecules in microvascular endothelial cells (compared with endothelial cells in large blood vessels) increases susceptibility to infiltration of inflammatory molecules, which can activate endothelial cells and diminish NO bioavailability.

In addition to intra‐individual comparisons across vascular beds (microvasculature vs. large blood vessels), we demonstrated that individuals with greater cardiorespiratory fitness better preserve microvascular endothelial function during hypoxic exposure. One of the well‐known benefits of cardiorespiratory fitness is an improved redox status (the balance between ROS production and antioxidant defence). Regular exercise and physical activity have been shown to increase antioxidant levels and reduce the production of ROS. In our study, we observed that those with superior cardiorespiratory fitness had the smallest hypoxia‐induced reduction in microvascular function, which might be attributable to a beneficial redox status and greater NO bioavailability in these individuals.

Early evidence for the role of oxidative stress and NO bioavailability in hypoxia‐induced endothelial dysfunction came from Bailey et al. (2013), who investigated blood oxygen saturation, redox status, NO bioavailability and endothelial function in lowlanders in three experimental conditions of increasing oxidative stress (normoxia, acute hypoxia and exhaustive exercise) and compared them with well‐adapted (healthy) and maladapted (chronic mountain sickness) high‐altitude residents. They identified that oxidative stress, measured by the presence of free radicals, was mildly elevated in adapted highlanders, but substantially increased in maladapted highlanders. Furthermore, maladapted highlanders exhibited worse hypoxaemia (lower oxygen saturations), which was accompanied by higher concentrations of free radicals, lower concentrations of antioxidants, lower nitrite bioavailability and worse vascular reactivity, with significant correlations between each of these variables. Our own work (Jones et al., 2021) is consistent with the original mechanism proposed by Bailey et al. (2013), but rather than comparing lowlanders and genetically distinct high‐altitude natives, we identified intra‐individual differences between vascular beds and inter‐individual differences within lowlanders. Our finding that superior cardiorespiratory fitness is associated with protection from hypoxia‐induced endothelial dysfunction begins to move the field on from the necessary work of characterizing the nature of hypoxic effects to identifying protective countermeasures and possible preventative interventions. However, we reported only a cross‐sectional correlation and did not conduct an experimental intervention.

Although the application in hypoxia is new, the idea that exercise training status can protect against endothelial dysfunction in physiological states of elevated oxidative stress is not new. Oxidative stress is generally increased with older age and has been implicated in the ageing process and the development of age‐related diseases. Indeed, >20 years ago, Taddei et al. (2000) used strain‐gauge venous occlusion plethysmography to investigate the combined effects of older age and exercise training status on the forearm blood flow response to acetylcholine with and without intravenous vitamin C. The age‐associated decline in response to acetylcholine was ameliorated by exercise training status in older adults. In addition, older sedentary participants showed age‐associated increased inhibition with l‐NMMA (NO inhibitor) that was ameliorated in older athletes. The administration of vitamin C, which donates electrons to neutralize ROS and other free radicals, corrected the l‐NMMA‐induced inhibition in older sedentary individuals but not in older athletes. Given that administering vitamin C does not improve endothelial function in populations with a ‘healthy’ redox status (young adults and older athletes), the observed vitamin C‐induced improvement in endothelial function supports the potential for targeted interventions to reverse the impairment in endothelial function specifically in the context of oxidative stress (e.g., in hypoxia).

1 **Connected Articles**

- Bailey, D. M., Rimoldi, S. F., Rexhaj, E., Pratali, L., Salinas Salmòn, C., Villena, M., McEneny, J., Young, I. S., Nicod, P., Allemann, Y., Scherrer, U., & Sartori, C. (2013). Oxidative‐nitrosative stress and systemic vascular function in highlanders with and without exaggerated hypoxemia. *Chest*, *143*(*2*), 444−451.
- Jones, D. T., Macdonald, J. H., Sandoo, A., Oliver, S. J., & Rossetti, G. M. K. (2021). The deleterious effects of acute hypoxia on microvascular and large vessel endothelial function. *Experimental Physiology*, *106*(*8*), 1699−1709.
- Stone, R. M., Ainslie, P. N., Tremblay, J. C., Akins, J. D., MacLeod, D. B., Tymko, M. M., DeSouza, C. A., & Bain, A. R. (2022). GLOBAL REACH 2018: intra‐arterial vitamin C improves endothelial‐dependent vasodilatory function in humans at high altitude. *The Journal of Physiology*, *600*(*6*), 1373−1383.

In a recent study, Stone et al. (2022) used the same methodology as Taddei et al. (2000) (strain‐gauge plethysmography) to investigate the effects of intravenous vitamin C administration on hypoxia‐induced endothelial dysfunction. The forearm blood flow response to acetylcholine decreased by ∼30% at high altitude (4300 m) compared with sea level but was largely restored with the addition of vitamin C infusion. Furthermore, consistent with the original work of Bailey et al. (2013), the magnitudes of both the reduction in endothelial function and the response to vitamin C were positively associated with the severity of hypoxaemia. In other words, those with the lowest oxygen saturations appeared to experience the greatest reduction in endothelial function that was explained by greater oxidative stress. Taken together, these papers support the notion that impairment of endothelial function in hypoxia is attributable, at least in part, to oxidative stress, and that oxidative stress might be a modifiable target for protection.

Bailey et al. (2013) highlighted those at exacerbated risk owing to this pathway, whereas our own work (Jones et al., 2021) identified individuals protected from vascular decline, possibly owing to this pathway, and subsequently, Stone et al. (2022) have built on this to demonstrate that targeted experimental manipulation of the pathway overcomes the impairment. Interventions to target each point of the pathway (hypoxaemia, oxidative stress and NO bioavailability) might protect against hypoxia‐induced impairment in vascular dysfunction both in lowlanders sojourning to high altitude and in high‐altitude natives. However, there is currently no evidence that supplemental antioxidants provide effective protection for vascular function. To date, studies using oral supplementation of antioxidant cocktails (e.g., 1 g of ascorbic acid, 400 IU of α‐tocopherol acetate and 600 mg of α‐lipoic acid) have not been consistently successful at improving oxygen saturations and, importantly, have not assessed endothelial function. Equivocal results in the efficacy of oral antioxidant supplementation to improve oxygen saturations might be attributable to different ascent profiles, dosages and intervention durations or to the method of administration (oral vs. intravenous). Supplements administered orally might not reach the endothelium, and intravenous injection is used as an acute experimental manipulation to investigate mechanisms; it is not proposed as a feasible prophylactic treatment. Indeed, Stone et al. (2022) are careful not to overstate whether vitamin C is a viable intervention (particularly if it requires intravenous injection). The evidence is not yet clear enough to provide practical recommendations, and future studies are needed to determine the feasibility and efficacy of specific antioxidant interventions. Although our own study implies that easily practicable interventions to improve sea‐level cardiorespiratory fitness might provide protection (Jones et al., 2021), we adopted a cross‐sectional approach. Experimental research is needed to confirm the efficacy of any practical recommendations relating to exercise training.

A consistent finding across all these papers is that hypoxia and other conditions associated with oxidative stress (ageing and a sedentary lifestyle) reduce endothelium‐dependent vascular reactivity (measured by FMD and response to acetylcholine), but do not affect endothelium‐independent vascular reactivity (measured by the NO donor glyceryl trinitrate or sodium nitroprusside). This is regardless of the method of assessment or the vascular bed being assessed (microvasculature or large vessels). Combined, this provides compelling evidence that it is NO‐dependent endothelial function that is impaired by hypoxia, and not the capacity of smooth muscle to respond to NO. These papers provide diverse evidence but consistently support the interpretation that the hypoxia‐induced impairment in endothelial function is, at least in part, caused by elevated oxidative stress and impaired NO bioavailability. Furthermore, individual differences appear to influence susceptibility to this effect; individuals experiencing worse hypoxaemia, worse oxidative stress and/or lower bioavailability of NO will experience greater impairments in vascular function. Methods to reduce oxidative stress and improve endogenous NO bioavailability (e.g., exercise training and antioxidants) might protect against hypoxia‐induced impairments in vascular reactivity. These findings have implications not only for those travelling or residing at high altitude, given that hypoxia is characteristic of several diseases at sea level (e.g., chronic obstructive pulmonary disease) and is implicated in the progression of cardiovascular disease, which itself exacerbates vascular hypoxia, resulting in a positive feedback loop. The research progression outlined in this article sets the scene for targeted interventions to protect against hypoxia‐induced endothelial dysfunction at high altitude and in pathological states.

## AUTHOR CONTRIBUTIONS

All authors have approved the final version of the manuscript and agree to be accountable for all aspects of the work. All persons designated as authors qualify for authorship, and all those who qualify for authorship are listed.

## CONFLICT OF INTEREST

None declared.

## FUNDING INFORMATION

No funding was received for the preparation of this article.

## References

[eph13387-bib-0001] Bailey, D. M. , Rimoldi, S. F. , Rexhaj, E. , Pratali, L. , Salmòn, C. S. , Villena, M. , McEneny, J. , Young, I. S. , Nicod, P. , Allemann, Y. , Scherrer, U. , & Sartori, C. (2013). Oxidative‐nitrosative stress and systemic vascular function in highlanders with and without exaggerated hypoxemia. Chest, 143(2), 444–451. 22922469 10.1378/chest.12-0728

[eph13387-bib-0002] Jones, D. T. , Macdonald, J. H. , Sandoo, A. , Oliver, S. J. , & Rossetti, G. M. K. (2021). The deleterious effects of acute hypoxia on microvascular and large vessel endothelial function. Experimental Physiology, 106(8), 1699–1709. 34036677 10.1113/EP089393

[eph13387-bib-0003] Stone, R. M. , Ainslie, P. N. , Tremblay, J. C. , Akins, J. D. , MacLeod, D. B. , Tymko, M. M. , DeSouza, C. A. , & Bain, A. R. (2022). GLOBAL REACH 2018: Intra‐arterial vitamin C improves endothelial‐dependent vasodilatory function in humans at high altitude. The Journal of Physiology, 600(6), 1373–1383. 34743333 10.1113/JP282281

[eph13387-bib-0004] Taddei, S. , Galetta, F. , Virdis, A. , Ghiadoni, L. , Salvetti, G. , Franzoni, F. , Giusti, C. , & Salvetti, A. (2000). Physical activity prevents age‐related impairment in nitric oxide availability in elderly athletes. Circulation, 101(25), 2896–2901. 10869260 10.1161/01.cir.101.25.2896

